# Altering One's Body-Perception Through E-Textiles and Haptic Metaphors

**DOI:** 10.3389/frobt.2020.00007

**Published:** 2020-02-18

**Authors:** Ana Tajadura-Jiménez, Aleksander Väljamäe, Kristi Kuusk

**Affiliations:** ^1^DEI Interactive Systems Group, Department of Computer Science and Engineering, Universidad Carlos III de Madrid, Madrid, Spain; ^2^Department of Psychology, Universidad Loyola Andalucía, Seville, Spain; ^3^UCL Interaction Centre, University College London, London, United Kingdom; ^4^School of Digital Technologies, Tallinn University, Tallinn, Estonia; ^5^Design Department, Estonian Academy of Arts, Tallinn, Estonia

**Keywords:** haptic clothing, e-textiles, multisensory body-perception, embodiment, virtual environments, experience design, vibration, tactile arrays

## Abstract

Technologies change rapidly our perception of reality, moving from augmented to virtual to magical. While e-textiles are a key component in exergame or space suits, the transformative potential of the internal side of garments to create embodied experiences still remains largely unexplored. This paper is the result from an art-science collaborative project that combines recent neuroscience findings, body-centered design principles and 2D vibrotactile array-based fabrics to alter one's body perception. We describe an iterative design process intertwined with two user studies on the effects on body-perceptions and emotional responses of various vibration patterns within textile that were designed as spatial haptic metaphors. Our results show potential in considering materials (e.g., rocks) as sensations to design for body perceptions (e.g., being heavy, strong) and emotional responses. We discuss these results in terms of sensory effects on body perception and synergetic impact to research on embodiment in virtual environments, human-computer interaction, and e-textile design. The work brings a new perspective to the sensorial design of embodied experiences which is based on “material perception” and haptic metaphors, and highlights potential opportunities opened by haptic clothing to change body-perception.

## Introduction

Fashion exists in different areas of people's lives and plays an important role in shaping consumer culture (Sassatelli, 2007). It connects the symbolic and aesthetic expressions with the cultural meanings that objects carry (Pan et al., 2015). Clothing in fashion attempts to balance two contradictory aims: it focuses on making ourselves more attractive while it also protects our modesty (Kawamura, 2005). It is a symbolic product, which meaning changes with time (Kaiser, 1996). Concurrently, the biosocial aspects of fashion have been largely understudied as compared to its aesthetical aspects (von Busch, 2018). While the outer layer of the garment or textile is meant for being exposed to the world outside of ourselves, there is also the inner, very intimate and hidden side of each textile/material. It exists only for the wearer, and it is in constant touch with our body. These two layers relate to the notion of having different body images for “self” and for the “others.” We might dress ourselves up to play a certain role or to fit into a specific context. Clothing influences the way we behave in social situations, representing a public image of one's self and reinforcing an individual's self-concept and self-confidence (Solomon, 1987). At the same time, self-comfort is one of the most important features of clothing—clothes are perfect if they do not irritate the wearer, if they are imperceptible, if they create pleasant sensations. Technology, however, could be used to enhance or amplify certain perceptions. Beyond the concept of “enclothed cognition” (Adam and Galinsky, 2012), that primarily focuses on the symbolic meaning and the physical experience of wearing clothes, e-textiles, defined as a type of fabric that contains electronic components, can be used to elicit different and augmented perceptions of one's body through clothing.

Neuroscience research has shown that body-perceptions, or the way people perceive their body appearance and their physical capabilities, are not something fixed. These body-perceptions keep changing continuously in response to sensory signals related to one's body (Botvinick and Cohen, 1998; Tsakiris, 2010; Tajadura-Jiménez et al., 2012). Research has also shown how these body-perceptions have an impact on the way people interact with the environment, as one needs to keep track of the configuration, size and shape of the different body parts when performing actions (Head and Holmes, 1911; Maravita and Iriki, 2004). Moreover, body-perceptions are at the basis of self-identity (Longo et al., 2008) and are also tightly linked to self-esteem (Carney et al., 2010) and social interaction. Further, recent neuroscientific studies have also shown that sensory feedback related to one's body can be used to alter body-perceptions (Sanchez-Vives and Slater, 2005; Haggard et al., 2007; Tajadura-Jiménez et al., 2012, 2015a, 2017a,b, 2018; Kurihara et al., 2013; Senna et al., 2014; Azañón et al., 2016; Radziun and Ehrsson, 2018). For instance, presenting discrepant visual and tactile cues, or visual and proprioceptive cues, about one's body can lead to change one's body-perceptions (e.g., perceiving and acting as if one's arm was longer) (Kilteni et al., 2012). More recently, research has also shown that sound feedback can be used to alter body-perception. For instance, one may get the perception of having a longer or a stronger arm, if when tapping one's hand on a surface the sounds produced are heard from a farther distance or stronger than expected, and this will also influence the subsequent arm movements and even one's emotional state (Tajadura-Jiménez et al., 2012, 2015b,c, 2016). Would it be possible to exploit such bottom-up multisensory mechanisms to design clothes that change body-perceptions?

While audio-visual cues have been dominating in feedback and communication technologies, tactile or haptic cues represent a good complementary if not the only channel in some cases, like in space or underwater scenarios (Brewster and Walker, 2000; Perret and Vander Poorten, 2018). Tactons or tactile icons, are structured, abstract messages that can be used to communicate messages non-visually (Brewster and Brown, 2004). New interfaces exploiting ultrahaptics enable new tactile surfaces by offering a mid-air haptic feedback development kit (Obrist et al., 2015; Shakeri et al., 2018; Ultrahaptics, 2018). Tactile sensations can be also delivered by electric stimulation and this has been used in rehabilitation of movement disabilities (Remotion, 2018). The Teslasuit concept is another example, where location based electric stimulation of the skin is fine tuned to the last element delivering haptic feedback straight to the whole body via a full body constume (Teslasuit, 2018).

A number of works have explored the integration of haptic feedback in clothing with various purposes. One of the pioneering works of using e-textiles for capturing and receiving sensations is the Cute Circuit's Hug Shirt from 2002 (CuteCircuit, 2018) that allowed users to send and receive physical hugs. In a similar vein, since 2000s MIT Media Laboratory has worked on the field of Psychohaptics (Vaucelle et al., 2006), exploring wearables supporting psychotherapy and touch-therapy protocols. On the other hand, there are proposals for clothing that measures and communicates visually to the outside world how the wearer feels. Philips design Bubble dress (El Fakih, 2011) and Sensoree Mood Sweater (Sensoree, 2012) do exactly that through shiny LED surfaces covered by textiles.

Haptic technologies are already entering the market but the potential of the sensory experiences they can deliver is still largely unexplored. In this paper we set to explore the possibility to design clothes that change body-perceptions through the use of tactile feedback. Vibrotactile patterns or tactons are increasingly used to convey recognizable messages (Ferguson et al., 2018). However, our aim was to design haptic patterns based on “material perception” as a way to elicit different body-perceptions. We were inspired by the work of Kurihara et al. (2013) where the feeling of being “robotized” is induced by vibration and sound accompanying the flexing of joints. We see such sensory stimulation as a way to create and explore enactive and cross-modal metaphors (Seitz, 2005).

We set to design a tactile feedback that could make the person feel as if made of a different material (e.g., rocks, water) to trigger body and emotional responses associated with such materials–could a rock link to feelings of being harder or stronger? would I feel more relaxed and happier if I am made of water?

**Figure 1:**
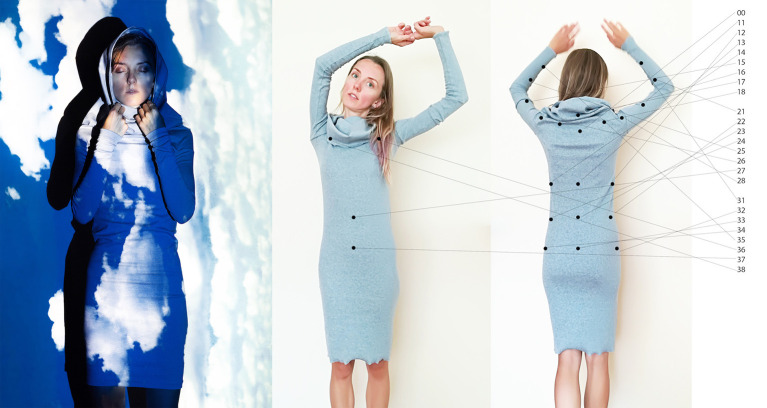
Altering one's body-perception through e-textiles. This figure represents our concept. Sensory-feedback integrated in the inner part of the garment (vibration motors and textile materials in play) changes the perceived body “material” via haptic metaphors (e.g., feeling as if my body would be made of rocks, water or “a cloud”), impacting on body-perception. The numbers on the right of the figure refer to the potential placement of 25 vibration motors on a tubular dress that would allow experiencing around the body the vibration patterns tested in Experiment 2–the numbers correspond to those used in Figure 4 to illustrate the distribution of the 25 motors used in Prototype 2. Video showing the project concept: https://vimeo.com/289294125. Written informed consent for the publication of this image and video has been obtained from the individuals appearing in them.

We adopted a novel approach in this work, which was framed as a science, technology and the arts (STARTS) collaborative project, bringing together expertise from art/design projects on changing people's behavior through textile design solutions and research on cognitive neuroscience (design process described in Kuusk et al., 2019). Our starting point was an exploration phase, in which we followed the Research through Design Methodology (Zimmerman et al., 2007; based on Frayling, 1993) in which designers perform research as they practice design to better ground their process. Drawing on the project's aim–altering people's body-perception though e-textile–various solutions were considered for providing tactile sensations from inside of a garment. Vibration motors, and especially the possibility of creating the sensation of moving touch on the skin through those, seemed having the greatest potential in terms of the effect delivered and the readiness of the off-the-shelf technology. A first rough prototype was created to communicate the idea of vibration moving along the garment's inside. It was a flat rectangle textile with a line of vibration motors sewn on it. This was the fastest way to have a tangible proof of concept in order to lead the discovery process further. By sensing the vibration moving on different parts of the body, we decided to build the first prototype (Figure 1) with more lines of vibration motors. This one could be used similarly on different parts of the body, but also to create a sleeve to try out the moving sensation when it surrounds a whole limb. When creating the prototype, we used the haptic metaphor of motion, like an insect moving through the surface of the body or being under the shower or under a waterfall. But how to create a sensation of air, water or other perceived substance flowing inside of one's body through such motion patterns?

Experiment 1 is a pilot study set to explore with our first prototype how vibration patterns could translate into specific bodily sensations and material sensations when experienced through the inside of the garment. We tested three different body areas (arm, center of the back, and upper back) with two different vibrotactile patterns in each area (moving outwards or inwards). Results from this pilot study showed that some material associations emerged from some of the vibrotactile patterns (e.g., air, water, sand, or rocks), as well as related bodily sensations. These associations related to the materiality helped us to build up Prototype 2 for Experiment 2, which allowed exploring a number of haptic metaphors bridging the materiality and sensations associated with them.

Based on the results from the pilot study, we created Prototype 2 to allow generating vibration patterns that were designed with three materials in mind, a “cloud,” “water,” and “rocks.” Our main hypothesis was that, apart from the vibration pattern, the textile surface between the skin and the motors also modulates the haptic sensation, and for that reason we chose two different textile surfaces to be tested. The aspect of different material surfaces influencing its perception has been earlier demonstrated (Hollies, 1989; Bergmann Tiest, 2010; Ramalho et al., 2013). We chose to build Prototype 2 to be touched with the hand, to make differences between conditions more noticeable, since face and hand areas representation in somatosensory cortex are richest manifesting its higher sensitivity as compared to other body parts (Nolan, 1982, 1985), and to address some limitations identified in Experiment 1. Experiment 2 is a controlled user study using Prototype 2 and set to investigate the interaction between the fabric chosen to embed the electronics (i.e., the vibration motors) when designing e-texiles and the vibration patterns themselves. In particular, in Experiment 2 we tested whether three vibration patterns–haptic metaphors–felt through two textile surfaces may translate into specific materiality and bodily sensations when touched with the hand.

## Experiment 1

### Materials and Methods

#### Participants

Ten participants [six females and four males, mean age 36.4, Age range 18–55] took part in the experiment. Participants gave their informed consent prior to their inclusion in the study. The study was conducted in accordance with the ethical standards laid down in the 1964 Declaration of Helsinki and approved by the Research Ethics Committee at Universidad Carlos III de Madrid (reference number: CEI n° 2018_004).

#### Stimuli Preparation

**Figure 2:**
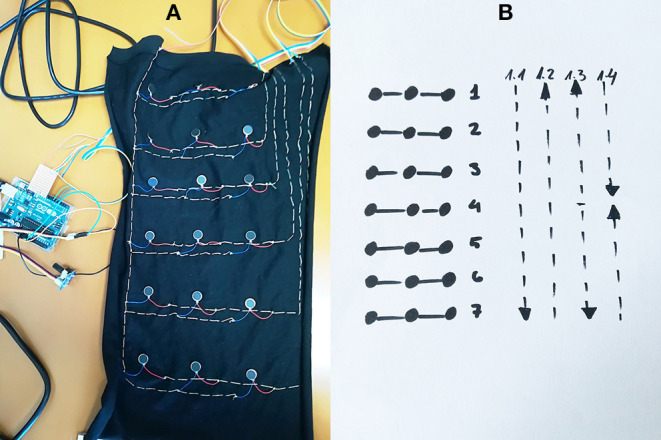
**(A)** Vibration motor positioning on the first *Magic Lining* prototype used in Experiment 1; **(B)** 3 × 7 vibrotactile array with numbered triplets and vibrotactile patterns' directions.

**Figure 3:**
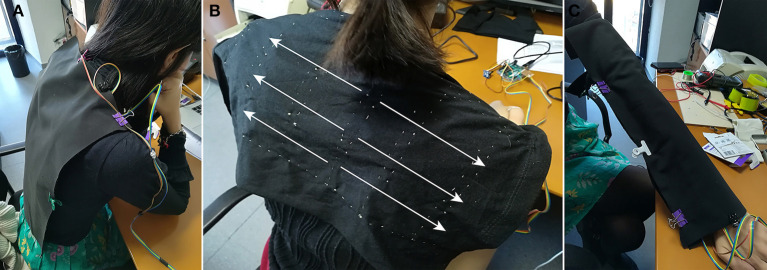
*Magic Lining* first prototype used in Experiment 1 worn by the study participant on **(A)** the upper back (U patterns); **(B)** the central back (C patterns; the image shows the expanding vibrotactile pattern C1.3); and **(C)** around the arm (A patterns).

Prototype 1 allowed generating vibration patterns that could potentially translate into bodily sensations when experienced through the inside of the garment. The system was comprised by 21 vibration motors (10 mm diameter, 2.7 mm thick, 2–5 V, 0.9 g weight) placed on jersey textile and connected with Shieldex 117/17 dtex 2-ply conductive thread. The motors were distributed forming a 3 × 7 rectangular matrix. Motors were separated from each other by 5 cm, and therefore the matrix occupied an area of 15 × 35 cm^2^ (see Figures 2, 3). The vibration motors were connected to an Arduino UNO linked to a computer running Arduino software. The software allowed generating different vibrotactile patterns.

#### Design

There were four vibrotactile patterns created by sequential activation of seven triplets (see Figure 2–right panel. The codes for creating the vibrotactile patterns are provided as Supplementary Material). Each 3-motor triplet vibrated for 200 ms (ON), with 0 ms delay between sequential triplets activation. A delay of 500 ms was introduced at the end of the sequence and each sequence was repeated 30 times. The four patterns were the following:

- Pattern 1.1 (“contraction”): lines 1 to 7 activated sequentially;
- Pattern 1.2 (“expansion”): lines 7 to 1 activated sequentially;
- Pattern 1.3 (“expansion”): lines 4, 3/5, 2/6, 1/7 activated sequentially;
- Pattern 1.4 (“contraction”): lines 1/7, 2/6, 3/5, 4 activated sequentially.

Three possible body locations were tested: arm (A), center of back (C), and upper back (U), see Figure 3. In the arm location the cloth was wrapped around the arm; in the back locations the cloth was opened and placed extended in the back, vertically in the center of the back location and horizontally in the upper back location. Though there were 12 possible combinations of location and pattern, we focused on testing the following six possible experimental conditions (three body locations with two vibrations patterns each), to test the effects of contraction and expansion:

- **A1.1**: a wave from the wrist to the upper arm (pattern 1.1);
- **A1.2**: a wave from the upper arm to the wrist (pattern 1.2);
- **C1.3**: a wave from the center of the back toward the upper and lower back (pattern 1.3);
- **C1.4**: a wave from the upper and lower back toward the center of the back (pattern 1.4);
- **U1.3**: a wave from the center of the upper back toward the shoulders (pattern 1.3);
- **U1.4**: a wave from the shoulders toward the center of the upper back (pattern 1.4).

Note than when defining what we meant by “contraction” and “expansion” we took the “center of the body” as our reference point: vibrations moved either away from this center of the body (outwards movement, or “expansion”) or approached the center of the body (inwards movement, or “contraction”). For the arm, we consider as “center of the body” the participants' body trunk (as opposed to the arm middle point) and therefore we chose patterns 1.1 and 1.2. For the patterns in the back, our reference point was the center of the back and therefore we chose patterns1.3 and 1.4.

#### Procedure

We used a within-subjects design to account for the great variability of bodily sensations between subjects (Tajadura-Jiménez et al., 2015a). The experiment was conducted in a dedicated space, with participants sitting in a chair and focused on doing the experimental tasks. Participants were informed that the aim of the project was to investigate the possibilities for altering the perception people have of their own body “through the garment's inside.” They were told we would place the material samples in their arm and back and that we wanted them to reflect on the different sensations these samples evoke on them. They were also informed that the experiment did not involve any risk for them, that they were free to withdraw from the study without penalty if they wished to, and that data acquired could be referred to in research papers, reports or conference proceedings albeit anonymously. After participants had been provided with task instructions, they were first asked to fill in a questionnaire that assessed their emotional state, body sensations and sensations of materiality. These responses were considered as the baseline response (Baseline condition). Next, they were equipped with the prototype and completed a set of six experimental conditions differing in the vibration pattern and body location used. These six conditions were presented in a randomized order and after each of them we quantified aspects of the experience by asking participants to fill in the same questionnaire used in the Baseline condition.

In this questionnaire, participants were asked to select a score that best expresses their subjective reactions during the experimental condition using 7-point Likert-type response items. This questionnaire was adapted from previous studies (Longo et al., 2008; Tajadura-Jiménez et al., 2015a), with some new items added in order to explore other possible bodily sensations elicited by the vibrations. The questionnaire was comprised by 15 statements, which ranged from: “I felt slow” to “I felt quick” (Speed); “I felt light” to “I felt heavy” (Weight); “I felt weak” to “I felt strong” (Strength); “I felt slouched” to “I felt up straight” (Posture); “I felt natural (as usual)” to “I felt unnatural” (Naturality); “I felt flexible” to “I felt stiff” (Flexibility); “I felt soft” to “I felt hard” (Hardness); “I felt insensitive” to “I felt sensitive” (Sensitivity); “I felt small” to “I felt large” (Size); “I felt cold” to “I felt hot” (Temperature); “I felt fit” to “I felt unfit” (Fitness); “I felt loose” to “I felt tense” (Tension); “I felt as if my body was not mechanical at all” to “…extremely mechanical” (Mechanic); “The feeling of my body is less vivid than normal” to “…more vivid than normal” (Vividness); “The feelings about my body are not at all surprising/expected” to “…extremely surprising/unexpected” (Surprise).

In addition, we asked participants to rate their emotional subjective reactions using three 9-point Likert-type response items, ranging from “Unhappy, Negative” to “Happy, Positive” (Valence); from “Unaroused, Calm” to “Aroused, Excited” (Arousal); and from “Submissive, slightly frightened, not in control” to “Dominant, important, in control” (Dominance) (Bradley and Lang, 1994).

Finally, as we were interested in exploring how the different vibration patterns could possibly translate into material sensations, after each condition we invited participants to report possible materials/elements that they felt that surrounded their body or that their body was made of when experiencing the different conditions. We provided participants with a set of elements that they could recognize from their environment and with distinctive perceptual qualities of density and hardness (including air, water, cotton, sand, oil, wood, rocks; see related work by Wongsriruksa et al., 2012). Nevertheless, we invited participants to report any other material/element related to their haptic experience. Note that our intention with this list was not to run statistical analysis on participants responses, but to inspire participants and help them in the process of thinking about materials, as this part of the testing was exploratory.

## Results

Questionnaire data were analyzed with non-parametric Wilcoxon tests comparing each pattern with the baseline condition. The alpha value was set to 0.05 for the statistical tests. Tables 1, 2 show the results for expansion (A1.2, U1.3, C1.3) and contraction (A1.1, U1.4, C1.4) patterns.

**Table 1:** Median (Range) scores for questionnaire data in Study 1 (7-level Likert items except for 9-level valence, arousal and dominance scales) and for the “expansion” patterns.

**Dependent variables**	**Baseline**	**Arm A1.2**	**Center C1.3**	**Upper U1.3**
Valence*	7 (4–8)	3.5 (3–8)* (*p* = 0.011)	6.5 (5–8)	7 (5–8)
Arousal	6 (3–7)	5.5 (3–7)	4.5 (2–7)	6 (2–8)
Dominance	5 (4–7)	5 (2–7)	5 (1–6)	5 (1–7)
Speed	4 (2–7)	4 (2–6)	4 (2–5)	3.5 (1–6)
Weight*	4.5 (3–6)	4 (2–5)* (*p* = 0.038)	4 (3–5)	3.5 (1–5)
Strength	5 (4–6)	4 (3–6)	4 (3–6)	4 (2–6)
Posture	4.5 (3–6)	5 (3–6)	4 (1–6)	4 (3–6)
Naturalness	4.5 (3–5)	5 (2–7)	5 (3–6)	5 (2–7)
Flexibility	4 (2–6)	4 (2–7)	4 (2–6)	4 (2–5)
Hardness	4 (3–5)	4.5 (1–6)	3.5 (2–6)	4 (1–6)
Sensitivity	5 (3–6)	5 (1–7)	5 (2–6)	5 (2–6)
Size	4 (3–6)	4 (1–6)	4.5 (3–6)	4 (2–6)
Temperature	4 (3–5)	4 (1–4)	4 (2–5)	4 (4–6)
Fitness	4 (3–6)	4 (3–5)	4.5 (4–6)	4 (2–5)
Tension	4 (3–6)	5 (2–6)	4.5 (3–7)	4 (1–6)
Mechanic*	3.5 (2–5)	5 (2–7)* (*p* = 0.017)	4 (2–4)	4 (2–7)
Vividness	4 (3–5)	4 (2–6)	4 (1–6)	5 (3–6)
Surprise*	3 (2–4)	4 (3–7)	5 (1–6)	5 (2–7)* (*p* = 0.014)

Bold font with *marks significant mean rank difference (p-value indicated) between the Baseline and the “expansion” conditions.

**Table 2:** Median (Range) scores for questionnaire data in Study 1 (7-level Likert items except for 9-level valence, arousal and dominance scales) and for the “contraction” patterns.

**Dependent variables**	**Baseline**	**Arm A1.1**	**Center C1.4**	**Upper U1.4**
Valence*	7 (4–8)	6 (4–8)	5 (3–7)* (*p* = 0.022)	5 (2–8)* (*p* = 0.019)
Arousal	6 (3–7)	6 (1–7)	5.5 (1–6)	5 (2–7)
Dominance	5 (4–7)	5.5 (1–7)	4 (3–7)	5 (3–6)
Speed	4 (2–7)	4 (2–5)	3 (1–5)	3.5 (2–4)
Weight*	4.5 (3–6)	3.5 (2–5)* (*p* = 0.038)	4.5 (3–7)	4 (3–6)
Strength	5 (4–6)	4 (3–5)	4 (3–5)	4 (2–6)
Posture*	4.5 (3–6)	4 (2–6)	3 (2–6)* (*p* = 0.026)	4 (2–6)
Naturalness	4.5 (3–5)	4 (2–6)	5 (2–6)	4.5 (2–6)
Flexibility	4 (2–6)	3.5 (3–5)	4.5 (3–6)	4 (3–6)
Hardness	4 (3–5)	4 (1–6)	4 (2–6)	4 (3–6)
Sensitivity	5 (3–6)	5 (3–7)	5.5 (3–7)	5 (2–6)
Size*	4 (3–6)	4 (3–6)	3 (1–4)* (*p* = 0.038)	4 (2–5)
Temperature	4 (3–5)	4 (4–6)	4 (3–4)	4 (3–5)
Fitness	4 (3–6)	4 (1–5)	4.5 (4–7)	5 (4–6)
Tension	4 (3–6)	4 (1–6)	4.5 (3–7)	5 (2–5)
Mechanic*	3.5 (2–5)	4 (2–6)	4 (1–6)	4.5 (2–6)* (*p* = 0.035)
Vividness	4 (3–5)	4 (3–6)	4 (1–5)	4 (2–5)
Surprise*	3 (2–4)	4 (3–6)* (*p* = 0.020)	5 (3–6)* (*p* = 0.006)	4 (2–6)

Bold font with *marks significant mean rank difference (p-value indicated) between the Baseline and the “contraction” conditions.

As shown in Table 1, during expansion patterns and as compared to the baseline condition, participants felt **less positive** for the arm-applied vibrations, i.e., pattern A1.2 (*z* = −2.54, *p* = 0.011). They felt **lighter** in A1.2 (*z* = −2.07, *p* = 0.038), with a similar trend for U1.3 (*z* = −1.90, *p* = 0.058). Further, participants felt their body as more **mechanic** in A1.2 (*z* = −2.39, *p* = 0.017) than in the baseline condition.

As shown in Table 2, during contraction patterns participants felt **less positive** in U1.4 (*z* = −2.34, *p* = 0.019) and C1.4 (*z* = −2.30, *p* = 0.022) than in the baseline condition. They felt **lighter** in A1.1 (*z* = −2.07, *p* = 0.038) than in the baseline condition. They also felt changes in **posture**, feeling less up straight in C1.4 than in the baseline condition (*z* = −2.23, *p* = 0.026). Further, participants felt **smaller** in C1.4 (*z* = −2.07, *p* = 0.038) and felt their body as more **mechanic** in U1.4 (*z* = −2.11, *p* = 0.035) than in the baseline condition.

Finally, participants overall found the vibration conditions more **surprising** than the baseline condition. There were significant differences between the baseline condition and A1.1 (*z* = −2.33, *p* = 0.020), C1.4 (*z* = −2.72, *p* = 0.006) and U1.3 (*z* = −2.45, *p* = 0.014). There were no significant differences in other scales (all *ps* > 0.05).

The exploratory questions of materiality helped us to understand that different vibrotactile patterns could induce different material associations. For instance, the patterns starting in the center of the back and moving outwards (C1.3 and U1.3) seemed to elicit sensations related to one's body being made of air (reported by four people for the condition U1.3), water (reported by five people for the condition C1.3 and by four people for the condition U1.3), sand (reported by four people for the condition U1.3) or rocks (reported by three people for the condition C1.3). For condition C1.3 participants also reported to feel “their body more fluid and malleable,” “relaxed” (two participants), “stronger and more confident,” and in condition U1.3 they reported “comfort,” “elevating, and concentrating in elevating more but without physical effort,” “like in a lake with a drop forming waves,” “relaxed,” “sensitive.” On the other hand, for the patterns moving inwards in the back (C1.4 and U1.4), for condition C1.4 participants reported feeling “heavy,” “very rigid and if I was made of concrete,” “like my body was being slightly pushed by some external force, out of my control,” “muscle tension,” and for condition U1.4 participants reported feeling “calm,” “pushed by someone and alert,” “like a concrete block with sand falling on top,” “tight muscles,” “excited” and “slightly anxious.”

For the arm patterns, for the pattern moving in the arm toward the wrist (A1.2) people reported as if their body was made of “something thick” (reported by four people), but also “tense,” “someone pulling my arm and forcing me to the world,” “very surprised,” “a bit uncomfortable,” “becoming mechanical and able to move heavy things, while simultaneously keeping relaxed,” “excited.” For the pattern moving in the arm from wrist to shoulder (A1.1) people reported feeling “comfort,” “self-focused,” “self-conscious,” “more relaxed,” “surprised, nice sensation” and that “muscles were growing.”

These exploratory questions helped to realize that different patterns seem to induce different material associations. For instance, the patterns starting in the center of the back and moving outwards seemed to elicit sensations related to one's body being made of air, water, sand or rocks; the pattern moving in the arm toward the wrist related to “something thick”. To have a closer relation to the materiality and sensations we created mind map for three concepts sensations: a “cloud,” “water,” and “rocks” (e.g., we described a “cloud” with words as cozy, cuddle, calm, air, soft, white, light (color), light (weight), flexible, fluffy, round, etc). The associations related to the materiality helped us to build up the next prototype.

Other learnings from the exploratory Experiment 1 were taken into account in the design of the new prototype and the controlled Experiment 2. First, for the back position, the prototype had to be very tight to the participants body or they reported not feeling some of the vibrators; sometimes we had to reposition the prototype to ensure they could feel all vibrators. This implied interaction between the participant and the experimenter, who had to touch the body of the participant to fit the prototype, and this could potentially have triggered some particular affective state in the participants. Further, it also made participants more aware of the electronic parts inside the fabric, as well as increased the duration of the experiment. Future versions of this prototype should be really tight and close to the body, potentially having different versions for different body sizes. For Experiment 2, we decided to stimulate the hands, as these would make differences between vibration patterns more noticeable, given their higher sensitivity as compared to other body parts (Nolan, 1982, 1985). Hands are also a relevant body part in relation to clothing–think for instance in gloves–and e-textiles are often experienced with the hands. Moreover, with the “hands” setup we eliminated the potential confound of the experimenter having to interact and touch the participants to place the prototype, which might have triggered some particular affective state in Experiment 1. In Experiment 2 participants themselves switched vibration patterns, so that the experiment could run smoothly, with shorter duration than Experiment 1, and with no interaction with the experimenter from the beginning to the end of the experiment.

## Experiment 2

### Materials and Methods

#### Participants

Nineteen participants [seven females; mean age 37.8, Age range 19–66 took part. Participants gave their informed consent prior to their inclusion in the study. The study was conducted in accordance with the ethical standards laid down in the 1964 Declaration of Helsinki and approved by the Research Ethics Committee at Universidad Carlos III de Madrid (reference number: CEI n° 2018_004).

#### Stimuli Preparation

**Figure 4:**
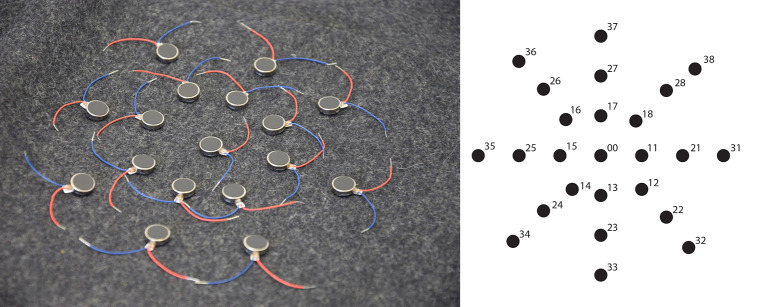
Prototype 2 used in Experiment 2 consisted of 25 vibration motors placed on felt material and connected with thin electric wires. The motors of Prototype 2 are distributed forming an octagon, resembling metaphorically a spider net. Video showing the project process: https://vimeo.com/272190793. Written informed consent for the publication of this video has been obtained from the individuals appearing in them.

Prototype 2 allows generating vibration patterns, which translate into materiality and bodily sensations when touched with the hand. Prototype 2 consisted of 25 vibration motors (10 mm diameter, 2,7 mm thick, 2–5 V, 0.9 g weight) placed on felt material and connected with thin electric wires. The motors were separated from each other by 1.5 cm, and therefore the octagon occupied an area of ~20 × 20 cm. The motors are distributed forming an octagon, resembling metaphorically a spider net (see Figure 4).

Based on the results from the exploratory pilot study (Experiment 1) we created mind maps that helped us to design three vibration patterns and to choose two textile surfaces (material samples) to deliver the sensations as close as possible to the following concepts, or, as first named in Stanney (1995), spatial haptic metaphors:

- **Water**, which to us also signifies flowing, wave, smooth, movement, cold;
- **Cloud**, which to us also signifies light, soft, warm, airy, fluffy, cozy, slow, calm;
- **Rocks**, which to us also signifies cold, stiff, polished, square, edgy, sharp, heavy.

**Figure 5:**
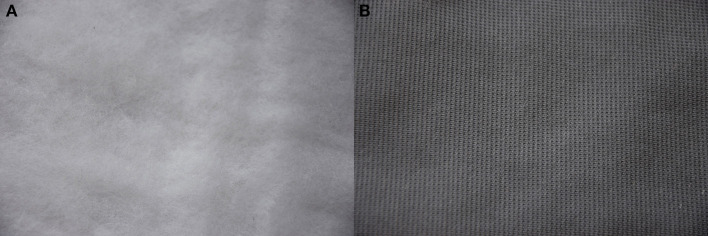
Textiles surfaces used in Experiment 2: **(A)** Material A was a fluffy soft white non-woven polyester, which is normally found inside warm jackets; and **(B)** Material B was a black structured woven (waffle) polyester, which is possibly used for light jackets, skirts, and pants.

When selecting the two textile surfaces, we took into consideration around 40 textile surfaces with different characteristics to evaluate their influence in sensations elicited by the vibration movements felt when touching them. We experienced each of the textile sample with the three patterns and selected the two that felt to us the most extreme from each other: textile surface 1 (Material A) was a fluffy soft white non-woven polyester, which is normally found inside warm jackets; and textile surface 2 (Material B) was a black structured woven (waffle) polyester, which is possibly used for light jackets and skirts, pants (see Figure 5).

**Figure 6:**
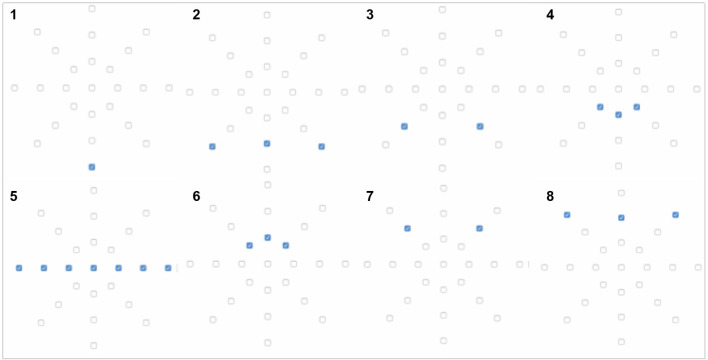
Pattern 1, “water” haptic metaphor, was created turning the vibration motors ON with current 100 mA and 1,000 ms steps (0 ms delay between steps) in the shown sequential order (steps 1–9).

**Figure 7:**
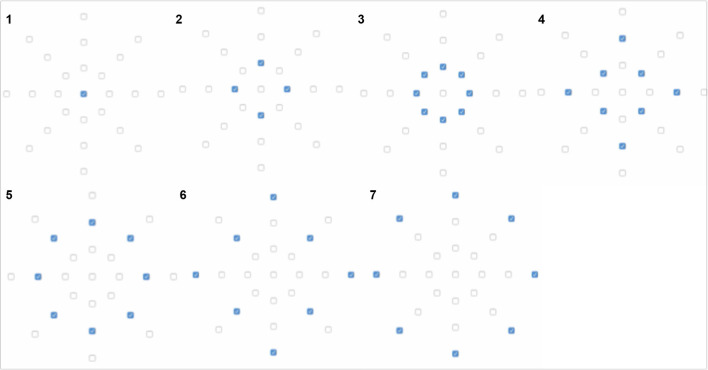
Pattern 2, “cloud” haptic metaphor, was created turning the vibration motors ON with current 120 mA and 400 ms steps (0 ms delay between steps) in the shown sequential order (steps 1–7).

**Figure 8:**
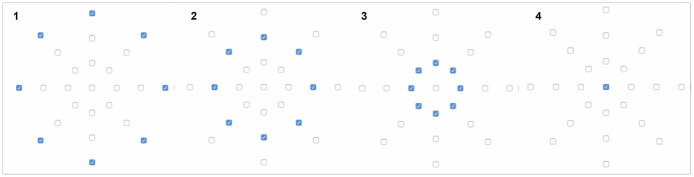
Pattern 3, “rocks” haptic metaphor, was created turning the vibration motors ON with current 200 mA and 200 ms steps (0 ms delay between steps) in the shown sequential order (steps 1–4).

**Figure 9:**
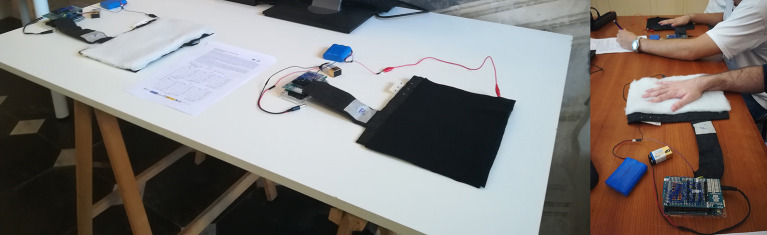
*Magic Lining* second prototype and set-up in Experiment 2. Participants across the different conditions placed their hand on the two material samples, once at a time, and experienced the three different patterns by pressing the three soft buttons in the top left corner of the prototype to switch pattern.

The vibration patterns were developed through first person perspective, “*where the movements, somatics and aesthetic sensibilities of the designer, design researcher and user are at the forefront”* (Höök et al., 2018), as described in the Research through Design methodology, reframing the problem through making the *right* thing (Zimmerman et al., 2007). We experimented with various “movements” of the vibration through different materials on our own bodies, reflected on that, changed them until we found a configuration that most resembled the chosen metaphors. When developing the vibration patterns, our design started from the haptic metaphors–the sensations we could expect when encountering these materials. Then, we ideated the vibrotactile patterns that would correspond to these sensations. We programmed the vibration movements and adapted them while feeling the sensations. For example, we changed the duration of the vibrations, the duration between the vibrations, the number of motors vibrating at the same time and the order of the vibration motors. Finally, we converged to three patterns, Pattern 1, Pattern 2, and Pattern 3, that had the characteristics of three haptic metaphors (as we perceived them)—see patterns and parameters varied in Figures 6–8 (the codes for creating the vibrotactile patterns are provided as Supplementary Material). Three soft buttons with numbers 1, 2, and 3 were added to the prototype to allow changing pattern (see Figure 9).

#### Procedure

As in the testing of Prototype 1 here we employed a within-subjects design, with three vibrotactile patterns (Patterns 1, 2, and 3) and two textile surface samples (Materials A and B). The experiment was conducted in a dedicated space, with participants sitting in a chair and focused on doing the experimental tasks. After participants had been provided with task instructions, they were invited to touch first one of the material samples and to experience the three different patterns in the order they wished to, and then switch to the second material sample and experience the three patterns in that sample (see Figure 9). Participants were free to change pattern at any given time, by pressing the soft buttons in the prototype. The order of presentation of the two textile surfaces samples was randomized between participants. Participants were not explicitly told that the patterns were the same in both material samples.

After each condition participants completed a questionnaire that assessed their emotional, bodily, and materiality sensations. Based on the lessons learnt in Experiment 1, we adapted the questionnaire used to become shorter and more focused on the variables of interest. In particular, to assess the emotional responses of participants, from the four items in the questionnaire of Experiment 1 (valence, arousal, dominance, and surprise) we chose the first two items, as these are the ones most commonly used to characterize emotions in a two-dimensional state (Bradley and Lang, 1994). From the other items in Experiment 1, we chose four items (weight, strength, hardness, and size) that could describe both material properties and properties of a person's body appearance or capabilities. Even though the items “posture” or “mechanic” showed significant differences in Experiment 1, we discarded them because they were either less relevant for the hand stimuli (i.e., posture) or because they could be ambiguously interpreted (i.e., mechanic for us meant “no fluid” but for some participants it meant being “robotized”). Therefore, the questionnaire used in Experiment 2 was comprised of six statements, which ranged from: “I feel unhappy” to “I feel happy” (Valence); “I feel calm” to “I feel nervous” (Arousal); “I feel light” to “I feel heavy” (Weight); “I feel soft” to “I feel hard” (Hardness); “I feel weak” to “I feel strong” (Strength); “I feel small” to “I feel large” (Size). For these statements, we asked participants to select a score that best expressed their sensations during the experimental condition using 5-point Likert-type response items. In addition, in order to test how the different vibration patterns could possibly translate into material sensations, we presented participants with a checkbox list of six possible materials/elements related to their experience, including the three ones guiding the experimental design (i.e., water, cloud, rocks). The full list was: water, cloud, concrete, sand, rocks and plastic (they could mark more than one material and also add further items to this list). The items selected represent different levels of hardness to softness, from solid to fluid. We chose materials with different characteristics: water and sand for us were both fluid and soft. Plastic is solid and hard but less than rocks or concrete, which were both solid and hard. We chose these words to make the “material” concepts in a way that everyone can understand them. This is, we decided not to use words as “polymer” but earthly concepts, so that the responses were not confounded by knowledge on material-specific language.

## Results

Note that five participants missed to respond to one of the items for one of the conditions–missing data was substituted by the round average of the other five conditions for that participant.

In order to investigate both the main effects and the interaction between the factors material sample (two conditions) and vibration pattern (three conditions) we conducted non-parametric factorial analyses on aligned rank transform (ART) data using ARTool (Wobbrock et al., 2011). The ART relies on a pre-processing step that “aligns” data before applying averaged ranks, after which common Analyses of Variance (ANOVA) can be applied. We conducted separated 2 × 3 ANOVAs on the aligned rank transform data of each questionnaire item, for which the alpha value for significance was set to 0.05. In case of significant main effects of vibration patterns, these were followed by *t*-tests between patterns, with the *p*-value adjusted with the recommended Tukey method for comparing a family of three estimates. Significant interactions between pattern and vibration were followed by interaction contrasts, which look at differences of differences, using the “testInteractions” function (Marascuilo and Levin, 1970; Boik, 1979), which is part of the R Phia module. The Holm method for *p*-value adjustment was used, as recommended.

**Figure 10:**
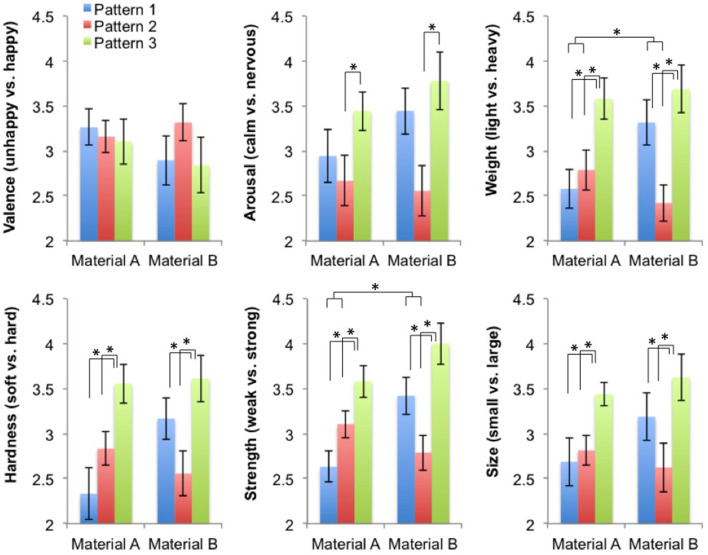
Mean (with Standard Errors) scores for questionnaire data in Prototype 2 testing (5-level Likert items). Haptic metaphors correspondence: Pattern 1–“cloud,” Pattern 2–“water,” and Pattern 3–“rocks”; Material A–soft surface, Material B–harder surface. *Marks significant mean differences between conditions and interaction effects.

For **Arousal**, there was a significant effect of vibration pattern [*F*_(2, 90)_ = 7.65, *p* < 0.001], but no effect of material sample or interaction between factors. As shown in Table 3 and Figure 10, independently of the material sample participants felt more aroused with the pattern 3 than with pattern 2 [*t*_(90)_ = −3.90, *p* < 0.001].

**Table 3:** Median (Range) scores for questionnaire data (5-level Likert items) in Study 2.

**Dependent variables**		**Pattern 1**	**Pattern 2**	**Pattern 3**
Emotional Valence	Material A	3 (2–5)	3 (2–5)	3 (1–5)
	Material B	3 (1–5)	3 (1–5)	3 (1–5)
Arousal	Material A	3 (1–5)	3 (1–5)	3.5 (2–5)
^*^VP (*p* < 0.001)	Material B	3 (1–5)	3 (1–5)	4 (1–5)
Weight	Material A	2 (1–4)	3 (1–4)	4 (2–5)
^*^VP (*p* < 0.001) M^*^VP (*p* = 0.012)	Material B	3 (1–5)	2 (1–4)	4 (1–5)
Hardness	Material A	2 (1–5)	3 (2–4)	4 (2–5)
^*^VP (*p* < 0.001)	Material B	3 (1–5)	3 (1–4)	4 (1–5)
Strength	Material A	3 (1–4)	3 (2–4)	4 (2–5)
^*^VP (*p* < 0.001) M^*^VP (*p* = 0.017)	Material B	3 (2–5)	3 (1–4)	4 (2–5)
Size	Material A	3 (1–5)	3 (2–4)	4 (3–5)
^*^VP (*p* < 0.001)	Material B	3 (1–5)	3 (1–4)	4 (2–5)

* VP marks significant mean differences of vibration pattern and M^*^VP marks a significant interaction between material sample and vibration pattern. P-values for significant effects are indicated.

For **Weight**, the effect of vibration pattern was significant [*F*_(2, 90)_ = 10.83, *p* < 0.001], with no effect of material sample. As shown in Figure 10, independently of the material sample participants felt heavier with vibration pattern 3 than with pattern 1 [*t*_(90)_ = −2.97, *p* = 0.011] and pattern 2 [*t*_(90)_ = −4.59, *p* < 0.001]. Results showed also a significant interaction between factors [*F*_(2, 90)_ = 4.59, *p* = 0.012], driven by the fact that the difference between patterns 1 and 2 was bigger for Material B than for Material A [$X_{\left(1\right)}^{2}$ = 9.14, *p* = 0.007].

For **Hardness**, the effect of vibration pattern was significant [*F*_(2, 90)_ = 10.63, *p* < 0.001], with no effect of material sample. As shown in Figure 10, independently of the material sample participants felt harder with vibration pattern 3 than with pattern 1 [*t*_(90)_ = −3.84, *p* < 0.001] and pattern 2 [*t*_(90)_ = −4.13, *p* < 0.001]. Results showed also a non-significant trend toward interaction between factors [*F*_(2, 90)_ = 2.77, *p* = 0.068], driven by the fact that the difference between patterns 1 and 2 was inverted from Material A to Material B, with pattern 1 leading to “harder” sensations in Material B [$X_{\left(1\right)}^{2}$ = 4.83, *p* = 0.08].

For **Strength**, the effect of vibration pattern was significant [*F*_(2, 90)_ = 10.73, *p* < 0.001], and there was a non-significant trend indicating some effect of material sample [*F*_(1, 90)_ = 3.23, *p* = 0.075]. As shown in Figure 10, independently of the material sample participants felt stronger with vibration pattern 3 than with pattern 1 [*t*_(90)_ = −3.78, *p* < 0.001] and pattern 2 [*t*_(90)_ = −4.21, *p* < 0.001]. Further, independently of the vibration pattern they tended to feel stronger with material sample B than with sample A. Results showed also a significant interaction between factors [*F*_(2, 90)_ = 4.27, *p* = 0.017], driven by the fact that the difference between patterns 1 and 2 was again inverted from Material A to Material B, with pattern 1 leading to “stronger” sensations in Material B [$X_{\left(1\right)}^{2}$ = 8.16, *p* = 0.013].

Finally, for **Size**, there was a significant effect of vibration pattern [*F*_(2, 90)_ = 8.86, *p* < 0.001], but no effect of material sample or interaction between factors. As shown in Table 3 and Figure 10, independently of the material sample participants felt larger with pattern 3 than with pattern 1 [*t*_(90)_ = −3.26, *p* = 0.004] or pattern 2 [*t*_(90)_ = −3.94, *p* < 0.001].

There were no significant differences in felt happiness (valence) between conditions (*p* > 0.05).

**Figure 11:**
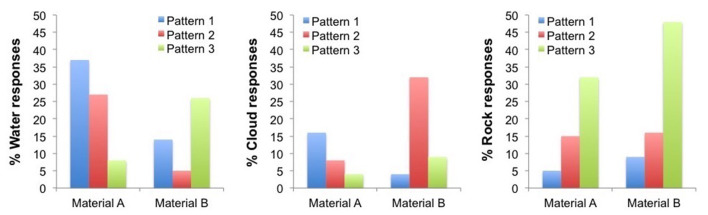
Percentage of “water,” “cloud,” and “rocks” reports for each of the six conditions. Haptic metaphors correspondence: Pattern 1—“cloud,” Pattern 2—“water,” and Pattern 3—“rocks”; Material A—soft surface, Material B—harder surface.

With regards to the materials/elements related to participants experience, Figure 11 shows that the highest percentage of “water” responses was given for Material A and vibration pattern 1 (37% responses), the highest percentage of “cloud” responses was given for Material B and vibration pattern 2 (32% responses) and the highest percentage of “rock” responses was given for Material B and vibration pattern 3 (48% responses), with high number of “rock” responses also observed for Material A and vibration pattern 3 (32% responses).

Responses to all material categories were distributed in each experimental condition as shown in Table 4. For Material A and vibration pattern 1, the highest number of responses was for “water” (37%), followed in percentage by “sand” (32%). For Material B and vibration pattern 1, the highest number of responses was for “concrete” (27%), followed by “sand” and “plastic” (both 23%). For Material A and vibration pattern 2, the highest number of responses was for “water” and “sand” (both 27%). For Material B and vibration pattern 2, the highest number of responses was for “sand” (37%), followed by “cloud” (32%). For Material A and vibration pattern 3, the “rock” responses (32%) were followed in percentage by “concrete” (28%). For Material B and vibration pattern 3, the “rock” responses got the highest percentage (48%).

**Table 4:** Responses to all material categories in Experiment 2.

**Material categories**	**Material sample A**			**Material sample B**		
	**Pattern 1**	**Pattern 2**	**Pattern 3**	**Pattern 1**	**Pattern 2**	**Pattern 3**
Water	37%	27%	8%	14%	5%	26%
Cloud	16%	8%	4%	4%	32%	9%
Concrete	0%	8%	28%	27%	0%	13%
Sand	32%	27%	8%	23%	37%	0%
Rocks	5%	15%	32%	9%	16%	48%
Plastic	10%	15%	20%	23%	5%	4%

## Discussion

The empirical results from testing Prototypes 1 and 2 show that various haptic metaphors induced by vibrotactile patterns within textile are possible, with impact on bodily sensations and emotional reactions. In other words, one can, speaking metaphorically, wear different sensations using e-textile. Experiment 1 was an exploratory pilot study set to test the potential of using contraction/expansion vibration patterns felt through the garment to elicit different body sensations. While we acknowledge that such exploratory approach is open to confounds, the results of Experiment 1 suggested that contraction/expansion vibrotactile patterns integrated in the garment's inside could be used to alter the perception of body posture, size, or weight among other bodily sensations, as well as to impact on emotional responses. Participants felt overall surprised by sensations elicited by vibrotactile patterns. The patterns on the arm and on the back were perceived differently with questionnaire data, which was corroborated by *post-hoc* interviews. For the back location, we saw that the contracting patterns created less positive sensations and feelings of being less up straight, smaller, and with a more mechanical body. On the contrary, the expansion patterns created the sensation of being lighter. For the arm location, participants reported being happier with the pattern moving from the wrist to the upper arm than the opposite (i.e., pattern moving toward the wrist); the latter made participants feel their body more machine-like. Different patterns also induced different material associations. For instance, the patterns starting in the center of the back and moving outwards seemed to elicit sensations related to one's body being made of air, water, sand or rocks; the pattern moving in the arm toward the wrist related to “something thick.” That brought us to the notion of haptic metaphors, which we tested in Experiment 2.

Experiment 2 was a controlled 3 × 2 study with three vibrotactile patterns and two material surfaces. The most important finding of this study is that it suggested that the textile surface texture itself interacts with the vibrotactile patterns in inducing different emotional reactions and/or bodily sensations and therefore should be taken into account when designing for haptic clothing. Vibration patterns influenced emotional arousal, and bodily sensations related to weight, hardness, strength and size. However, the material samples alone also influenced the perceived body strength and significantly interacted with vibration patterns in building sensations of hardness and strength. The interaction found between vibration and material sample implies that the effects found are not only the result of a change in vibration intensity when using different surface fabrics, but that they relate to the different associations or haptic metaphors elicited by the combination of both vibration and material surface. Further, this interaction shows that the association with a materials/concept such as a “cloud” is not achieved only by using a fabric which may visually resemble “a cloud” or which is soft (Material A), but by a combination of a material surface with a specific vibration pattern. Indeed, we do not find such “cloud” association with Material A, which one could argue that resembles more a “cloud” as it is a fluffy soft white material. We observe the “cloud” association for vibration pattern 2 with material sample B, but not for other patterns with the same fabric. These results suggest that all senses interact in building “haptic metaphors.” A possible alternative explanation is that the interaction effect found could be driven alone by the vibration pattern of the modulated spectrum of the fabric-covered vibrator, independently of the associations elicited by the material surface. Future studies should disentangle the underlying cause by conducting a comparison of our current conditions with the vibration patterns experienced through a material sample vs. a synthesized vibration pattern where intensities and frequencies are adjusted to reproduce the joint effect of the current vibration pattern experienced through the material sample [see for instance the related studies on the perception of visual surfaces were different stimulus variables were systematically varied in a series of psychophysical experiments (Gibson, 1950)].

Thus, results from Experiment 2 highlight the importance of considering haptic metaphors (i.e., water, cloud, rocks) as sensations in experience design for body-perceptions (e.g., being heavy, strong) and emotions. We showed that participants did give more reports of water for pattern 1, cloud for pattern 2 and rocks for pattern 3, matching the concepts that guided our design (Figure 11). Importantly, the sensations we associated to each of these haptic metaphors during the design process were also reflected in the sensations elicited in participants. For example, with the material sample 1 and with pattern 2 (which we chose thinking of “cloud”) participants felt calmer than with material sample 2 and pattern 3 (which we chose thinking of “rocks”). With the latter participants also felt heavier, harder, stronger and bigger than with the other conditions.

Beyond the specific results shown by the studies, this work was set to bring a new perspective on the design of technologies able to change body-perception by using e-textile. Our work resonates well with the developments in somaesthetics research domain and applications related to designing for various bodily experiences, such as (Shusterman, 2008; Höök et al., 2016) by exploring haptic metaphors that can lead to different bodily perceptions. Today, we see similar technology being used in concert performances and museum settings for allowing people to experience music with their bodies (CuteCircuit, 2016). Thanks to a science-art transdisciplinary collaborative project, which invited to think out-of-the-box, the idea of using haptic metaphors based on “material perception” emerged. Throughout the process we discovered, similarly to Satomi and Perner-Wilson (Satomi and Perner-Wilson, 2007), that e-textiles development can be very tailored. Every project is a new invention, using the knowledge from previous work, but always solving new challenges in new ways. In Experiment 2 we focused on sensations felt on the hand as this would make differences between conditions more noticeable, given the higher tactile sensitivity of hands as compared to other body parts (Nolan, 1982, 1985). Hence, future work should investigate the transferability of our results and concept from hand to whole body tactile stimulation and to the inner part of clothing, as well as investigating the potential use of electrocutaneous stimulation. In Figure 1 we represent the concept of a tubular haptic dress where the vibrations are placed so that the vibrotactile movement sensations elicited via haptic metaphors (i.e., the sensations associated with illusions of one's body being made of a different material, such as rocks, water or “a cloud”), would be the most vivid for the wearer. In this dress, the original “spider net” pattern would be placed on the whole body, and the placing of the vibration motors could follow the logic that it would be possible to experience movements over both of the arms as well as around the body. Since the vibrators are placed on the outer side of the arms, it is able to embrace the wearer and move around her. The cut and material of the dress shown in Figure 1 were selected based on the following criteria. For the vibrations to be felt as intended, the garment needs to be tight and close to the body. At the same time, the dress has to be flexible to allow the wearer to move freely. A jersey tubular dress with tight sleeves would allow both. However, not only needs the garment fit the wearer's body and allow a range of movement. The dress has to trigger sensations pleasant or inspiring to the wearer. Everything from the physical garment to the placement of the vibration motor arrays, to the programming has to be tailored for the specific use and user. As advertised by Teslasuit, the personalization of patterns is probably the key of such haptic clothing (Teslasuit, 2018).

From the perspective of the future work, we have a number of directions that deserve exploration. First, apart from questionnaires, objective measures should be used for assessing participants' sensations, such as changes in electrodermal activity related to arousal (Boucsein, 2012) or changes in touch behavior (e.g., velocity or pressure) which can be related to different sensations (e.g., Tajadura-Jiménez et al., 2014, 2015a,b). It may be interesting to test individual differences related to previous experiences with vibration gear (e.g., used for massage) and the persistence of the effects with longer exposure times or more repetitions. Second, multisensory prototypes where vibrotactile stimulation is paired with sound (or other sensory feedback) is a very promising direction where auditory-haptic metaphors could be more easily created using minimalistic tactile stimulation (e.g., cross-modal filling-in effects as in Väljamäe and Soto-Faraco, 2008 and references therein). Another multisensory pairing could be related to chemosensing and chemosignalling (Semin and de Groot, 2013). Third, our prototypes were only used for delivering sensations; however, closed-loop bio- or neurofeedback systems could also be possible. For example, in the recent project Bisensorial, a neuroadaptive vibroacoustic therapeutic device, used music and vibrotactile stimuli applied to the user's back to induce desired mental states (Maranan, 2017). Fourth, while the haptic clothing is a personal device, using this in social settings and combining it with other sensors (e.g., a magnetic compass in Nagel et al., 2005) creates countless possibilities for different social interaction scenarios, from passive, like in a new type of cinema, to active, as in a participatory theater. Finally, our work contributes to the fields of teleoperation and embodying new bodies and objects (e.g., Hohwy and Paton, 2010), expanding the notion of one's current physical body when having a subjective feeling of being “robotic” or “fluid” as in Kurihara et al. (2013), and also to limb prosthetics (Wijk and Carlsson, 2015), where full body haptic metaphors may help as a novel sensory substitution strategy.

The work makes us wonder about the fashion of the future. Is it something that has to be “experienced” rather than seen? How would the future catwalk be like? Would it be possible to understand what the model feels while “wearing the sensations”? Could we instead of wearing the newest cuts and patterns of famous fashion designers, wear the emotions they design? And could we download the desired emotions or feelings directly to our second skin?

## Data Availability Statement

All datasets generated for this study are included in the article/Supplementary Material.

## Ethics Statement

Written informed consent was obtained from the individual(s) for the publication of any potentially identifiable images or data included in this article.

## Author Contributions

AT-J, AV, and KK developed the study concept and contributed to the study design. Testing and data collection were performed by KK and AT-J. AT-J performed the data analysis and interpretation. AT-J, AV, and KK drafted, revised, and approved the final version of the paper for submission.

### Conflict of Interest

The authors declare that the research was conducted in the absence of any commercial or financial relationships that could be construed as a potential conflict of interest.

## Supplementary Material

Data Sheet 1 Experiment 1 Data.

Data Sheet 2 Experiment 2 Data.

Data Sheet 3 Codes for creating the vibrotactile patterns.
